# Does osteoporosis predispose falls? a study on obstacle avoidance and balance confidence

**DOI:** 10.1186/1471-2474-12-1

**Published:** 2011-01-03

**Authors:** Ellen Smulders, Wim van Lankveld, Roland Laan, Jacques Duysens, Vivian Weerdesteyn

**Affiliations:** 1 Sint Maartenskliniek, Research, Development and Education, P.O. box 9011, 6500 GM, Nijmegen, the Netherlands; 2 Sint Maartenskliniek, Department of Rheumatology, P.O. box 9011, 6500 GM, Nijmegen, the Netherlands; 3 Radboud University Nijmegen Medical Centre, Department of Rheumatology, P.O. box 9101, 6500 HB, Nijmegen, the Netherlands; 4 Research Center for Movement Control and Neuroplasticity, Department of Biomedical Kinesiology, Katholieke Universiteit Leuven, Leuven, Belgium; 5 Radboud University Nijmegen Medical Centre, Nijmegen Centre for Evidence Based Practice, Department of Rehabilitation, P.O. box 9101, 6500 HB, Nijmegen, the Netherlands

## Abstract

**Background:**

Osteoporosis is associated with changes in balance and physical performance and has psychosocial consequences which increase the risk of falling. Most falls occur during walking; therefore an efficient obstacle avoidance performance might contribute to a reduction in fall risk. Since it was shown that persons with osteoporosis are unstable during obstacle crossing it was hypothesized that they more frequently hit obstacles, specifically under challenging conditions.

The aim of the study was to investigate whether obstacle avoidance ability was affected in persons with osteoporosis compared to a comparison group of a community sample of older adults.

**Methods:**

Obstacle avoidance performance was measured on a treadmill and compared between persons with osteoporosis (n = 85) and the comparison group (n = 99). The obstacle was released at different available response times (ART) to create different levels of difficulty by increasing time pressure. Furthermore, balance confidence, measured with the short ABC-questionnaire, was compared between the groups.

**Results:**

No differences were found between the groups in success rates on the obstacle avoidance task (p = 0.173). Furthermore, the persons with osteoporosis had similar levels of balance confidence as the comparison group (p = 0.091). The level of balance confidence was not associated with the performance on the obstacle avoidance task (p = 0.145).

**Conclusion:**

Obstacle avoidance abilities were not impaired in persons with osteoporosis and they did not experience less balance confidence than the comparison group. These findings imply that persons with osteoporosis do not have an additional risk of falling because of poorer obstacle avoidance abilities.

## Background

Osteoporosis is a disease which is characterized by a decrease in bone mass density and a disruption of the normal trabecular architecture, which reduces the bone strength. The estimated prevalence of osteoporosis in the Netherlands is 5% in men and 17% in women aged 55 and over [1]. This is comparable with the prevalence of osteoporosis in the USA [2]. Because of their lower bone strength persons with osteoporosis have a higher risk of fall-related fractures. There are, however, several osteoporosis-related factors that may further add to the risk of fractures because of their effect on the risk of falling.

One such factor is fear of falling. Fear of falling is known to be related to a decrease in physical and mental performance, and to higher risk of falling [3,4]. In a study by Sinaki (2005), persons with osteoporosis reported significantly more fear of falling, measured with the Falls-Efficacy Scale (FES), than healthy controls [5]. Previous studies have shown that in women with osteoporosis or low bone mass increased fear of falling is associated with more falls[6], and balance confidence is related to measures of balance and mobility [7].

Another factor that contributes to the risk of falling relates to vertebral fractures, which are quite common in persons with osteoporosis. The prevalence of vertebral fractures is 25% in Caucasians aged 70 and over [8]. The prevalence is likely to be even higher because these fractures can occur without pain and are therefore not always diagnosed [9]. Vertebral fractures cause a change in body posture due to increased kyphosis of the thoracic spine, which is associated with decreased muscle strength in the back and lower extremities [5,10]. Furthermore, the change in posture causes a forward displacement of the centre of mass of the trunk, which imposes greater demands on balance recovery following a disturbance [5,11]. Indeed, increased kyphosis is related to postural instability [12], which is demonstrated by an increased postural sway [13,14], and the use of different balance strategies in persons with osteoporosis compared to healthy controls [13].

In addition to these deficits in postural balance during quiet standing tasks, persons with osteoporosis have also been reported to have reduced dynamic stability when crossing an obstacle during walking [5]. This challenging gait task is of great interest since most falls occur during walking, and tripping over obstacles is one of the most common causes of falls [15,16]. In previous research it was shown that elderly persons have reduced responses with later onset when stumbling over obstacles [17]. From this it was concluded that deficits in obstacle avoidance skills in elderly persons may represent an important fall risk. For this reason an obstacle avoidance task has been developed and was tested on young and elderly subjects [18,19]. The results clearly demonstrated a deterioration in obstacle avoidance skills with age [19]. Furthermore, it was shown that older recurrent fallers were less successful in avoiding obstacles than non-fallers [19]. It may be hypothesized that, due to their dynamic instability, persons with osteoporosis are also more prone to hitting obstacles and, consequently, to falls in daily life. However, in the study of Sinaki et al. [5], it was not reported whether the persons with osteoporosis failed more often in avoiding the obstacle than did the healthy controls. Furthermore, a disadvantage of studying obstacle avoidance performance during walking over ground is that participants adopt a slower more conservative gait before obstacle crossing[20], which contributes to increased body sway. Therefore, there is a need to study success rates in obstacle avoidance skills in persons with osteoporosis, while they walk at a fixed velocity.

The aim of this study was to investigate whether obstacle avoidance ability is affected in persons with osteoporosis compared to a community sample of older adults (comparison group). Since introducing a time constraint is known to magnify disease-related impairments [21-23], a procedure were participants walk at a fixed velocity and have to avoid sudden obstacles is recommended. Furthermore, we examined whether persons with osteoporosis experience more fear of falling, by measuring their balance confidence, and we investigated the relationship between balance confidence and obstacle avoidance performance. In this way, we aimed to gain further insight into the mechanisms that may contribute to an increased fall risk in persons with osteoporosis.

## Methods

### Participants

The participants of this study were community-dwelling older persons (n = 85) of at least 65 years of age with confirmed osteoporosis. All participants experienced at least one fall in the previous year and were able to walk 15 minutes without the use of a walking aid. Osteoporosis had to be diagnosed on the basis of a Dual Energy X-ray Absorptiometry (DXA) measurement (T-score ≤-2.5 at the femoral neck or lower back) either in the past or at the start of the study. Exclusion criteria were ophthalmic disorders, severe cardiac, pulmonary and musculoskeletal disorders, as well as neurological and/or orthopaedic pathologies associated with a high fall risk (e.g. stroke, Parkinson's disease or Rheumatoid Arthritis). The patients were enrolled from a larger study on the efficacy of a falls prevention program for persons with osteoporosis (trial registry number: NCT 00432692 (clinicaltrials.gov)) [24].

Data from the community sample of elderly persons (n = 99) had been collected in a previous study using the same experimental methods [19]. For this study the same in- and exclusion criteria were used, except that persons with confirmed osteoporosis were excluded from participation, however, no DXA-measurements were conducted at inclusion.

This study was approved by the medical ethical committee of the region Arnhem-Nijmegen and all participants gave written informed consent for participation

### Obstacle avoidance task

**Figure 1:**
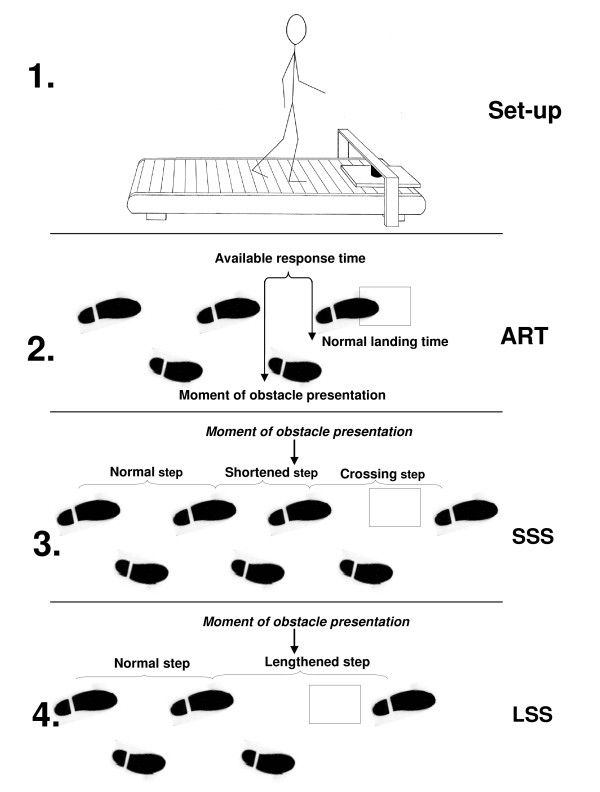
**Experimental set up and schematic explanation of some key concepts**: (Figure 1.1.) Experimental set up. In front of the treadmill a bridge with an electromagnet is placed. The obstacle is attached to the magnet and will be released at different available response times. (Figure 1.2.) Available response time (ART): the time between obstacle presentation and the predicted (unaltered) landing time of the foot. (Figure 1.3.) Short Step Strategy (SSS): additional foot contact is made in front of the obstacle prior to the actual crossing manoeuvre. (Figure 1.4.) Long Step Strategy (LSS): the step during which the obstacle is presented is lengthened to cross the obstacle. Figure 1 is adapted from: Schillings AM, Van Wezel BMH, Duysens J. J Neurosci Meth 67:11-17 and Den Otter AR, Geurts AC, de Haart M, Mulder T, Duysens J. Exp Brain Res 161:180-92.

The participants were instructed to avoid obstacles while walking on a treadmill (ENRAF Nonius, Type EN-tred Reha) at a fixed velocity of 3 km/hr, wearing comfortable shoes. This velocity was selected because it is known to be a comfortable walking speed for healthy older persons [25]. The participants were secured by a safety harness, attached to the ceiling. Above the front of the treadmill a bridge was placed with an electro-magnet. The obstacle (40 cm [length] × 30 cm [width] × 1.5 cm [height]) was held by the magnet in front of the left foot (Figure 1). The participants were asked to walk at a fixed distance of 10 cm to the obstacle. Two reflective markers were attached to the shoe on the left heel and hallux and a third marker was attached on top of the obstacle. Marker positions were recorded by a 6-camera 3 D motion analysis system (Vicon, 100 Hz). Before the experiment started the participants were given the opportunity to familiarize to treadmill walking and after that five practice trials of obstacle avoidance were performed.

Obstacle release was triggered by a computer (Weerdesteyn et al, 2005[19]). To determine the correct moment of obstacle release, the heel marker position was processed in real-time during the experiment. Algorithms were used to predict the next heel contact on the basis of the preceding steps. Based on this information the exact timing of obstacle release was determined. The obstacle was not released before a regular walking pattern had been achieved, which was defined as less than 50 ms difference in stride duration between two consecutive strides.

During the experiment, the obstacle was released at different phases of the gait cycle. to create different levels of difficulty. In the used protocol, an important determinant for success in the obstacle avoidance task is the Available Response Time (ART). The ART is defined as the time between obstacle detection and the estimated moment of foot contact with the obstacle. When time pressure increases (shorter ARTs) more failures are made [26-28]. The ART's are shorter for obstacles that are released near the end of the gait cycle. The experiment consisted of two series of 15 trials, in which the level of difficulty was randomly divided of the trials. The number of strides between two successive obstacle releases was variable so that the moment of obstacle release was not predictable.

The primary outcome measure was the obstacle avoidance success rate. During the experiment failures were noted by two observers. They were defined as contact of the foot with the obstacle. In case of disagreement the 3 D recordings were checked to verify whether the foot had touched the obstacle. The success rate was determined by dividing the number of successful trials by the total number of trails. Consistent with the methods used in a previous study in the community sample of older adults [19], success rates were calculated for ART categories of 200-250 ms, 250-300 ms, 300-350 ms and more than 350 ms.

As a secondary outcome on the task, we determined the strategy used to avoid the obstacle. Two strategies were used to avoid the obstacle. During a short stride strategy (SSS) the obstacle was avoided by shortening the stride before crossing and then crossing the obstacle in the next stride. During a long stride strategy (LSS) the obstacle is crossed by a lengthened stride. Prior research has demonstrated that an SSS is used in the majority of trials with short ARTs, whereas in the trials with long ARTs the LSS prevails [19,28,29]. This is due to the subjects' tendency to minimize the displacement of the foot from its original landing position [27]. Our study was designed such that, based on this minimal displacement criterion, the expected strategy to avoid the obstacle was an SSS in 50% of the trials and an LSS in the other half of the trials.

### Balance confidence

The short version of the Dutch translation of the Activity-specific Balance Confidence (ABC) scale was used to determine balance confidence [30-32]. The short-ABC has proven to be a valid and reliable measure which assesses balance confidence rated for 6 activities of daily living with a minimum score of 0% (no confidence) and a maximum score of 100% [33]. The mean score over the items was used in the statistical analysis.

### Statistical analysis

The overall success rates over the four different ART categories were compared between the two groups by means of a multilevel mixed-effects linear regression model.

From previous research it is known that increasing age negatively associated with obstacle avoidance success rates with an average decline of 1.5% per year [19]. The effect of sex on the obstacle avoidance task is not reported before, but both variables were included in the model to account for possible confounding. Furthermore, it was analysed whether these variables affected group outcomes (effect modification) by including the interaction effects 'Age' × 'Group' and 'Sex' × 'Group' in the model.

Strategy distribution and the scores on the short-ABC were compared between the persons with osteoporosis and the comparison group by means of an univariate general linear model, with 'Group' as fixed factor and 'Strategy' or 'ABC-score' as dependent factor. 'Age' and 'Sex' were also included in these analyses as covariates.

To test whether the level of balance confidence was of influence on the success rate on the obstacle avoidance task, the variable 'ABC-score' was also included in the regression model as confounder and as interaction factor with 'Group'.

For the analysis STATA.10 and SPSS12.0.1 were used. The level of α was set on 0.05.

## Results

The characteristics of the participants are described in Table 1. The persons with osteoporosis were slightly younger than the comparison group and used more medication. Furthermore, the group of persons with osteoporosis consisted of more females than the comparison group. The number of falls in the three months prior to the study was not different between the groups.

**Table 1:** Baseline characteristics of the participants

	*Osteoporosis (n = 85)*	*Comparison (n = 99)*
Age (years (mean (sd))*	71.0 (4.8)	73.7 (5.6)
Male:female*	5:80	23:76
Falls (% in prior 3 months)^¶^		
*0 fall*	71.8	67.5
*1 fall*	23.5	22.5
*> 1 falls*	5.7	10
Number of medications used (mean(sd))*	2.34 (1.67)	1.19(1.44)
*Bisfosfonates (%)*	68	-

*p < 0.01

*^¶^*Falls were recorded in the 3 months prior to the obstacle avoidance task by means of monthly fall registration cards.

### Obstacle avoidance success rate and strategies

'Age' and 'Sex' were identified as confounders for the success rates on the obstacle avoidance task (p ≥ 0.01). Higher age negatively influenced the success rates, and men had higher success rates than women in this study. The effects of age and sex were not different between the groups, as indicated by no interaction effect (respectively p = 0.494 and p = 0.268).

**Figure 2:**
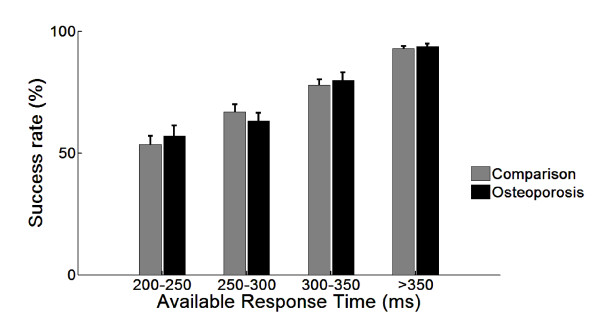
**Success rates per ART category (mean and standard error of the mean)**.

In Figure 2, the results of the obstacle avoidance task on success rate are presented. As expected the success rates increased when time pressure reduced (higher ARTs). In most ART categories the persons with osteoporosis had lower success rates, except for the ART category of 250-300 ms. Overall, the comparison group had approximately 3% higher success rates, but this difference was not significant (p = 0.173).

The total number of LSS and SSS were comparable between the groups (p = 0.822). The persons with osteoporosis used an LSS in 74% (SD = 14) of the trials, compared to 74% (SD = 20) in the comparison group. Age and sex had no influence on the choice of avoidance strategy (respectively p = 0.302 and p = 0.907).

### Balance confidence

Of the participants with osteoporosis one person did not complete the questionnaire correctly and one questionnaire was not returned. In the comparison group eight questionnaires were not returned or completed incorrectly. 'Age' and 'Sex' had an effect on the balance confidence scores; persons with higher age had lower scores (p = 0.001), and men scored higher on the ABC-questionnaire than women (p = 0.001). The mean score on the short-ABC questionnaire was 55.0 (SD = 19.6) in the group of persons with osteoporosis, and 59.7 (SD = 16.9) for the comparison group. There was no significant difference in balance confidence between the groups (p = 0.091).

Furthermore, balance confidence did not seem to influence the success rates of the participants, since 'ABC-score' was not identified as confounder (p = 0.148) nor was there an interaction effect between 'ABC-score' and 'Group' (p = 0.145).

## Discussion

The aim of this study was to investigate obstacle avoidance performance in persons with osteoporosis in comparison with a community sample of elderly persons. Persons with osteoporosis are known to be more unstable when crossing an obstacle [5], and to have deteriorated physical performance skills [5,7,10,12-14]. Therefore, it was hypothesized that their obstacle avoidance success rates would be lower. In contrast to previous studies that all demonstrated impaired obstacle avoidance performance in a variety of musculoskeletal and neurological conditions [21-23,34], our group of persons with osteoporosis did not have lower success rates compared to those of a community sample of elderly persons. This was even true for the most challenging obstacles (i.e. those with the lowest ARTs), in which disease-related impairments are generally most pronounced. Furthermore, both groups avoided the obstacle in a similar manner, as indicated by their comparable proportions of long and short step strategies.

In an earlier study (Sinaki et al. [5]) obstacle avoidance performance was investigated in kyphotic persons with osteoporosis compared to healthy subjects during walking overground with a fixed obstacle. They found that persons with osteoporosis had more body sway while avoiding obstacles than the control group. The functional consequences of the observed instability remained unidentified since the number of obstacle hits was not reported in the paper. It is very likely, however, that the participants hardly ever contacted the obstacle, because previous studies that used a similar set-up with a fixed obstacle reported the near-absence of any failed crossing attempts [35-37]. This can be explained by the fact that in such a set-up the obstacle can be detected well in advance, which implies that the available response time is high and avoidance success rates will be large. In our study we increased the time pressure in order to increase the probability of failures, thereby creating a more realistic daily life situation. Although the data collected in the present experiment did not allow us to calculate dynamic stability measures, as was done in the study by Sinaki et al., the results show that none of the potential group differences increased the risk of hitting the obstacle in the persons with osteoporosis, which was even true for the most challenging obstacles.

An advantage of our set-up was that it allowed us to control for potential osteoporosis-related differences in gait speed [38]. Since we tested the participants' obstacle avoidance abilities on a treadmill at a fixed velocity of 3 km/h, the between-group comparisons in the present study were not confounded by potential differences in gait speed.

In our analyses we also aimed to identify whether lower balance confidence scores associated with poorer performance on the obstacle avoidance task. In other studies it was found that higher levels of fear of falling resulted in decreased physical functioning [4,7,39], but a similar association could not be demonstrated in the present study. This could be due to the presence of the safety harness, which may have eliminated any unfavourable effects of fear of falling on obstacle avoidance performance in the high-anxious persons. Consequently, it was possible to truly test obstacle avoidance skills in persons with osteoporosis without fear of falling as co-determinant.

In agreement with other studies [19,28,40] our study confirmed the effect of aging on obstacle avoidance performance, with advancing age resulting in more failures. Therefore, age was included in the statistical analysis model, hereby also correcting for the small difference in age between the groups at baseline. A second finding, which was not previously reported, was that obstacle avoidance performance was different between the sexes, with women failing more often than men. A possible explanation for this sex-related difference may be associated with the observation that our participants used an LSS in the majority of the trials, which required them to lengthen the stride in order to avoid the obstacle successfully. Since the size of the obstacle was the same for all participants, the required lengthening of the stride might have been relatively smaller for men, who generally have longer legs than women.

The observation that both groups of older participants from our study preferred the LSS over the SSS for avoiding the obstacle is also in agreement with previous work [19,28]. Based on the minimal displacement theory, that states that the main criterion to choose a strategy is based on minimisation of displacement of the foot from its original landing position, a 50-50 distribution of strategies would be expected [27,28,41]. Young adults have indeed demonstrated equal proportions of LSS and SSS [19,28]. In our study, however, both groups used an SSS in only ~25% of the trials. The preference for the LSS in older persons may be based on safety considerations. It has been suggested that an SSS imposes greater demands on dynamic stability since it induces larger Centre of Mass (COM) disturbances. Additional research is necessary, however, to identify the causes of changes in the distribution of avoidance strategies with advancing age.

Another aim of the present study was to investigate whether persons with osteoporosis have more fear of falling than their comparison group. It might be expected that they are more afraid of falling because of their higher risk for injuries, but this has not yet been thoroughly investigated. In this study, there was no difference in balance confidence between the persons with osteoporosis and their comparison group, which indicates that they are not more fearful. In comparison with other studies, however, the average balance confidence scores were rather low for both groups of participants [30,33]. This is probably due to the inclusion of persons with a fall history, which is known to be associated with increased fear of falling [3].

A limitation of the present study was that, due to the use of a previously collected data set in the community sample of older adults there were some baseline differences between the groups in age and proportions of men and women. These relatively small differences were corrected for in our statistical analyses. A further difference between the groups concerned the number of medications used, with the persons with osteoporosis using on average 1.15 more medications than the comparison group. This difference can mainly be attributed to their use of bisphosphonates, which were taken by a majority of the participants with osteoporosis (Table 1).

Another limitation of this study was that we used a historic cohort of a general sample of the Dutch elderly population as comparison group. However, the study procedures and in- and exclusion criteria were identical. A disadvantage of this cohort was that some characteristics of the participants could not be obtained. For instance, at inclusion these persons had not been screened for the presence of osteoporosis (e.g. by means of a Dual Energy X-ray Absorptiometry (DXA)). Therefore, it cannot be ruled out that some of these persons did have undiagnosed osteoporosis. In the present study for persons with osteoporosis, those who applied for participation and met all our inclusion criteria except that they had not been diagnosed with osteoporosis before, were screened with the use of a DXA measurement as a last step of the inclusion procedure. Of these persons, 13% were diagnosed with osteoporosis. Since for both groups of participants (osteoporosis and comparison group) we used the same inclusion criteria and recruitment methods, it is expected that a similar number of persons with osteoporosis may have been present in our comparison group.

The present study could not confirm the presence of an osteoporosis-related fall risk factor related to obstacle avoidance performance. Further research is needed to identify osteoporosis-specific deficits in motor functioning and their potential contribution to the risk of falling in order to develop efficient interventions for the prevention of falls and injuries. A recent review showed that exercise interventions might reduce falls, fall-related fractures, and several risk factors for falls in individuals with low bone mass density. These exercise interventions should be, at least partly, weight bearing and include balance exercise and muscle strengthening exercises to reduce fall and fracture risk [42]. However more research is needed to investigate the effects of exercise on falls and fracture incidence in individuals with low BMD.

## Conclusions

In conclusion, the present study demonstrates that obstacle avoidance abilities were not impaired in persons with osteoporosis, since their success rates and avoidance strategies were comparable to those of a community sample of elderly persons. These findings imply that persons with osteoporosis do not seem to have an additional risk of falling because of poorer obstacle avoidance abilities. Furthermore, persons with osteoporosis did not experience more fear of falling than the comparison group

## List of abbreviations

**ART**: Available Response Time (the time between obstacle release and the estimated moment of foot contact with the obstacle if no adjustment of the stride had been made (Chen et al., 1994)); **ABC**: Activity-specific Balance Confidence; **BMD**: Bone Mineral Density; **COM**: Centre of Mass; **DXA**: Dual Energy X-ray Absorptiometry; **FES**: Falls-efficacy scale; **LSS**: Long Stride Strategy; **SSS**: Short Stride Strategy; **SD**: Standard Deviation.

## Competing interests

The authors declare that they have no competing interests.

## Authors' contributions

ES, WL, RL, JD and VW conceived and designed the study. ES, VW and RL recruited the participants, ES and VW performed the experiments and the analysis. ES made the first draft of the manuscript. WL, RL, JD and VW contributed to the analysis and interpretation of the data, and revised the manuscript critically. All authors read and approved the final manuscript.

## Pre-publication history

The pre-publication history for this paper can be accessed here:

http://www.biomedcentral.com/1471-2474/12/1/prepub
